# Care management staff perspectives on stigma and barriers to substance use treatment experienced by latine adults who use substances

**DOI:** 10.1016/j.dadr.2025.100342

**Published:** 2025-05-10

**Authors:** Christina S. Lee, Erika G. Cordova-Ramos, Damaris J. Rohsenow, Kim T. Mueser, Christine A. Pace, Rosemarie Martin, Suzanne M. Colby, Victoria Lopez, Melanie Morris, Jake R. Morgan, Ari Kriegsman, Mari-Lynn Drainoni

**Affiliations:** a Boston University School of Social Work, USA; b Department of Pediatrics, Boston University Chobanian & Avedisian School of Medicine, USA; c Evans Center for Implementation and Improvement Sciences, Department of Medicine, Boston University Chobanian & Avedisian School of Medicine, USA; d Center for Alcohol and Addiction Studies, Brown University School of Public Health, USA; e Center for Psychiatric Rehabilitation, Departments of Occupational Therapy and Psychological & Brain Sciences, Boston University, USA; f Department of Medicine, Boston University Chobanian & Avedisian School of Medicine, USA; g Department of Health Law Policy & Management, Boston University School of Public Health, USA; h Department of Behavioral Health, Mercy Medical Center, Boston, MA, USA; i Section of Infectious Diseases and Evans Center for Implementation and Improvement Sciences, Department of Medicine, Boston University Chobanian & Avedisian School of Medicine, USA

**Keywords:** Structural stigma, Intersectionality, Substance use, Latine, Primary care

## Abstract

**Background:**

Stigma related to substance use or addiction contributes to health care inequality. Structural stigma - embedded in societal conditions, policies, practices, and cultural norms - has been less studied than interpersonal (e.g., provider bias) and individual level stigma processes. The perspectives of staff working with patients who navigate health care systems can help to identify substance use stigma at the structural and interpersonal levels. The study aimed to examine staff perceptions of structural and interpersonal stigma processes, their association with barriers to substance use disorder (SUD) care, the interplay between different levels of stigma, and their impacts at the individual level.

**Methods:**

Care management staff (n = 20, 75 % community health workers, CHWs) from a complex care management program were interviewed about the challenges Latine compared to non-Latine patients faced in accessing care for substance use treatment. Thematic analysis was used to analyze interview transcripts. The Structural Stigma framework was used to guide analysis.

**Results:**

Structural and interpersonal stigma processes as well as intersectional stigma were associated with barriers to SUD care. Latine patients were reported as being frequently affected by intersecting systems of oppression due to multiple stigmatized identities (e.g., persons with substance use and as immigrants) than non-Latine patients. Structural and interpersonal stigma processes were associated with self-stigma and hindered help-seeking behaviors.

**Conclusion:**

Care management staff offer unique perspectives into how stigma at multiple levels is experienced by patients and perpetuated. Stigma processes may discourage the initiation of needed substance use care among Latine persons experiencing oppression.

## Highlights

• Community health workers are poised to identify stigma experiences and solutions.
• Structural stigma for Latines may be based on immigrant, substance use identities.
• Self-stigma and intersectional stigma discourage Latines from seeking SUD care.
• Solutions are needed to reduce the effects of all forms of stigma in health care.

## 1 Introduction

**Fig. 1:**
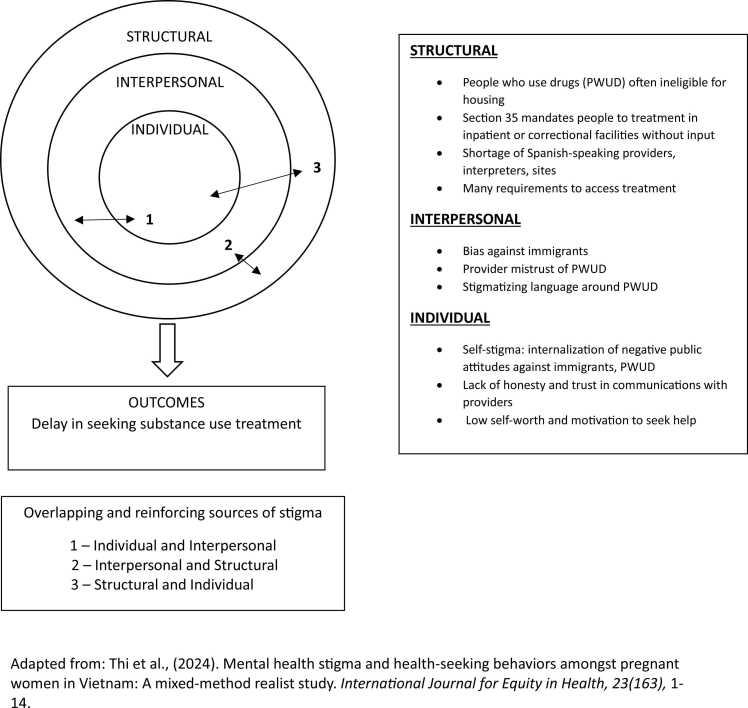
Individual, interpersonal, structural stigma, and barriers to substance use treatment.

Adapted from: Thi et al. (2024).

Substance use stigma, defined as the devaluation of persons with substance use disorders (SUD), hinders help seeking and recovery (Morris and Schomerus, 2023) (Kilian et al., 2021). Stigma involves negative stereotypes that foster low status and social exclusion (Carrara et al., 2023) and occurs at structural (policies, laws), interpersonal (interactions), and individual/intrapersonal (responses) levels (Carrara et al., 2023). Structural stigma refers to the “societal-level conditions, cultural norms, and institutional policies that constrain the opportunities, resources, and well-being of the stigmatized” (Hatzenbuehler and Link, 2014, p.1). Interpersonal stigma focuses on interactions between the “stigmatized” and the “stigmatizer,” (Hebl and Dovidio, 2005), and, along with individual stigma, has dominated most research (Earnshaw and Chaudoir, 2009, Hatzenbuehler, 2016, Link and Phelan, 2001, Logie et al., 2021, Smith et al., 2016). While often studied separately, interpersonal and structural stigma are interconnected, as the former can occur in the context of the latter (Evans-Lacko et al., 2015; Pachankis et al., 2014, Pachankis et al., 2015; Hatzenbuehler and Link, 2014; Hatzenbuehler and Pachankis, 2021). Emerging research suggests that structural stigma can influence individual-level responses, including health behaviors (e.g., Pachankis et al., 2014). Investigating stigma processes occurring at different levels can illuminate the pathways through which they contribute to individual behaviors among persons who use drugs (PWUD) (see Fig. 1).

Studies of structural substance use stigma are uncommon. Among 59 studies examining substance use stigma, only six directly mentioned structural stigma (Davis et al., 2022, Knaak et al., 2022, Livingston et al., 2012, Spata et al., 2024, Xin et al., 2023). Structural stigma may be less studied because of its complexity, abstract conceptualization, and occurrence within and across institutions (Adkins-Jackson et al., 2021). It can also occur outside of peoples’ awareness (Adkins-Jackson et al., 2021). Of three studies on stigma towards persons who inject drugs (Biancarelli et al., 2019, Motavalli et al., 2021, Paquette et al., 2018), only one (Paquette et al., 2018) referred to “macro-level” stigma, including only negative perceptions by health care professionals. No direct examples of structural stigma were provided. When participants mentioned societal issues that marginalize PWUD in healthcare, they did not frame these as structural stigma (Madden et al., 2024).

Intersectional stigma (Bowleg, 2012) refers to overlapping forms of stigma tied to multiple identities or experiences, so that individuals experience discrimination and marginalization in more than one way (Layland et al., 2022). Prejudices can exacerbate negative judgments (Bowleg, 2012, Cole, 2009). In one study, 900 adults showed greater implicit stigma towards PWUD, especially when the individuals were Latine rather than White (Kulesza, 2013). Latine adults demonstrate lower rates of initiation and utilization of SUD treatment than non-Latine adults (Mulia et al., 2014, Pinedo et al., 2018, Schmidt et al., 2006). One plausible hypothesis is that Latine individuals who use substances face greater barriers to accessing SUD treatment than non-Latine individuals due to the societal stigmatization of immigrants (Morey, 2018). Immigrant stigma influences all Latine individuals, including those born in the U.S. (Wang, 2018) because of racial profiling (Armenta, 2017, Noe-Bustamante et al., 2021, Pinedo et al., 2022, Szkupinski Quiroga et al., 2014, Viruell-Fuentes et al., 2012).

Qualitative methods can give shape to the “practices and policies that create, sustain, and reinforce health inequities” and suggest connections between structural stigma and individual outcomes (Adkins-Jackson et al., 2021, p. 542). Interviewing those who work with stigmatized populations offers a complementary perspective that can identify stigma at all levels. This study presents findings from interviews with staff working with Latine and non-Latine patients in a primary-care based complex care management (CCM) program, including community health workers (CHWs), who share community ties and lived experiences with the patients they work with, including exposure to structural inequalities. Program staff bring unique insights into how society might stigmatize patients within interconnected systems (Adkins-Jackson et al., 2021).

The current study used an intersectionality informed approach (Bowleg, 2012) guided by the Structural Stigma framework (Hatzenbuehler, 2016). Study primary aims were to explore perspectives on factors influencing SUD service delivery. We examined structural and interpersonal stigma processes, their association with barriers, intersectional stigma, and the effects of these stigma processes on the individual.

## 2 Methods

### 2.1 Study context

This study is a sub-study of a clinical trial (Clinicaltrials.gov NCT#04771650) examining the delivery of SUD services to Latine primary care patients enrolled in Complex Care Management (CCM) embedded within primary care practice. CCM was designed to coordinate care for the highest-risk patients in the Boston Medical Health System’s participating Medicaid accountable care organizations. Each patient was assigned a community health worker (CHW) and nurse team, with ancillary clinical staff such as social workers, housing specialists, and pharmacists called upon as needed. CHWs focused on patient outreach and engagement, health and social system navigation, care coordination, and addressing social needs such as transportation, housing, and food (NIH, 2014). Nurse care managers provided health education, chronic disease support, and direct coordination with medical providers. Social workers assisted with care coordination and connecting patients to social services. Each CCM team worked with a panel of 45 patients to address patient-directed goals, such as chronic disease self-management, obtaining resources, and accessing services, including SUD treatment. Approximately 65 % of patients in the CCM program had a SUD diagnosis and 19.6 % of all CCM patients identified as Latine.

### 2.2 Procedures

A semi-structured interview guide (see Supplemental Material) was created and refined by three members of the research team (CL, MLD, EGCR), reviewed by two panels of experts (addiction researchers, CCM physician leaders), modified, and then reviewed again by all members of the research team. The first author trained research assistants (RAs) on using the interview guide and probing participant responses. All three interviewers were first or second-generation immigrant Latina women. Their identities and lived experiences included navigating structural inequality and working with Latine adults in healthcare settings. All were committed to acknowledging potential biases that differed from their own. After practice together, each RA roleplayed with the first author. Once proficient in probing, recruitment began.

Participants were recruited in all CCM roles, including clinical, administrative and management, to obtain a variety of different and important perspectives. An email was sent to all CCM personnel from the CCM Medical Director and the study Principal Investigator. Participants provided verbal informed consent. Given the small sample size, participants were asked about their role and profession, but not other information, to protect confidentiality. Interviews (40–60 minutes) were conducted in English via Zoom or telephone from November 2021– February 2022 and audio-recorded. Participants were compensated with a $40 gift card to local stores. The study methods adhered to the Standards for Reporting Qualitative Research (O’Brien et al., 2014) and were approved by the hospital and university Institutional Review Boards.

### 2.3 Data analysis

Interviews were professionally transcribed, then analyzed using thematic analysis (Braun and Clarke, 2006) and Hatzenbuehler's (2016). Transcripts were coded using NVivo software [NVivo QSR International, 2021]. Two RAs (V.L., M.M.) met to discuss the study’s theoretical framework, reviewed codes, and discussed coding discrepancies to reach consensus. Once all the data were coded, they were grouped into the categories of Structural, Interpersonal, and Individual stigma.

To generate themes, codes were summarized using an interpretative descriptive approach (Thorne et al., 1997, Thorne et al., 2004) using existing clinical and experiential knowledge to offer in-depth interpretation in a contextualized way (Dobischok et al., 2023, Thorne et al., 2004). Alternative explanations were sought to broaden preliminary interpretations (Thorne et al., 2004). Triangulation was also used to enhance rigor; themes were cross-checked by individuals from different disciplines. After themes were first created, the first author reviewed them with co-author EGC-R, then with the entire research team. The themes were then reviewefd with CCM physician leaders, an example of peer debriefing, in which a peer not directly involved in the research process reviewed and discussed findings and interpretations with the research team to ensure that the research findings were grounded in CCM staff experiences (Creswell, 2013, Lincoln and Guba, 1985). Differences in interpretation were discussed by the research team until agreement was achieved. This process helped achieve an interpretation of findings that reflected various disciplinary perspectives.

## 3 Results

Of the 43 CCM personnel invited to participate, n = 20 (47 %) completed interviews: 15 CHWs, and 5 included nurses, social workers, and managers. This is reasonably representative of program staff which was 50 % CHWs, 40 % nurses, and 10 % social workers and staff in management roles. In the analysis, we identified three themes (see below). Quotes are provided in the tables, with speakers identified as CHW or non-CHW, along with an identifier to preserve anonymity. Quotes, including nomenclature and potentially stigmatizing terms, were presented unedited.

### 3.1 Theme 1: structural and interpersonal stigma processes were associated with barriers to SUD treatment

Laws and policies around accessing SUD treatment reflected substance use stigma at the structural level (Table 1, Theme 1). CCM staff noted that people with SUD were often ineligible for housing. They referred to the Section 35 law, which allows courts to commit a person with substance use involuntarily to treatment if they are a danger, as “incredibly traumatizing.” In Massachusetts, people are mandated to inpatient or correctional facilities for SUD treatment without having any say in their placement. CCM staff described onerous requirements for accessing and staying in SUD treatment, such as the need for daily in-person attendance. Structural stigma against immigrants was embedded in laws that restricted access to health care and reflected a lack of investment in resources. CCM staff noted a shortage of Spanish-speaking providers, interpreters, and treatment sites offering care in Spanish.

**Table 1:** Comments on structural and interpersonal stigma processes and their association to barriers.

Theme	Comment
1.Structural and interpersonal stigma processes were associated with barriers to SUD treatment	“For SUD patients, there’s the **social determinants like housing** being huge.” (HQ1008, CHW)
	“I think that methadone, the **amount of oversight, and policies needed to get methadone** makes it not a very attractive treatment option for a lot of people, especially if you’re trying to work or have other things in your life. It’s very proscriptive…” (HQ1003, non-CHW) “One of my Latinx patients is living at his parents’ on their couch. He tells me **he is afraid to access services because he doesn't want them to know** that he's using again.” (HQ1012, CHW)
	“I would say that from my previous experience my clients that identify as Latina/Latino/Latinx speaking about their level of comfort with providers **not understanding their language, not understanding their cultural needs**.” (HQ1007, non-CHW)
2.CCM staff indicated that Latine patients experienced intersectional stigma at structural and interpersonal levels more often than Latine patients, increasing barriers to care	“**Latinx patients often have more barriers** than the average CCM client. Some **don’t have health insurance.** It can be an immigration issue or they don’t qualify for MassHealth. It’s the top barrier. It’s on top of their substance use disorder. **There are other barriers that prevent Latinx patients from getting good health and getting into other programs.”** (HQ1004, CHW)
	“One of my patients has a criminal background and a dialysis patient and called the federal website number for substance abuse help, over 19 places, and was told no by each one. **Another person who is both of these [criminal and substance use] and not of a different culture got something prescribed to him right away for alcohol use.** I found that a little perplexing that one patient could go right to their provider within a day, while for this other patient – we’ve been waiting him for over two weeks.” (HQ1010, non-CHW)
3.Stigma processes at the structural and interpersonal levels reinforced each other and influenced individuals’ responses	“I’ve actually seen (Latine) patients **leave treatment** because they know they have a court date coming up. I’ve also had a couple of clients sitting around like ‘**I’m just a drunk that’s all I’ll ever be’** It’s the constant shame that’s placed on them”. (HQ1011, non-CHW)
	“It takes more to bring Latina women into any kind of help (for SUD) because they have **kind of taken in that shame**.” (HQ1029, CHW)

At the interpersonal level, CCM staff shared that Latine patients encountered prejudicial attitudes in health care settings. One staff member reflected that many Latine patients experienced being judged as “lazy,” “not wanting to change,” or “not willing to work” if they were in pain and/or unable to work. Others were seen as “stupid” because of their accent, and that providers often thought “less” of them. Staff described interactions between patients and workers or providers in health care and legal settings that reflected mistrust of PWUD, such as not providing adequate pain medication or being skeptical of their motivation for seeking help. This stigma overlapped with discrimination against Black, Indigenous, or People of Color patients, who were regarded as “malingerers” when they sought SUD care**.**

### 3.2 Theme 2: CCM staff indicated that Latine patients experienced intersectional stigma at structural and interpersonal levels more often than non-Latine patients, increasing barriers to care

CCM staff noted that many opportunities to engage in society (e.g., employment) and health insurance coverage were unavailable to PWUD with a criminal justice history (Table 2, Theme 2). Among PWUD with criminal justice involvement, Latine persons were more vulnerable to sources of intersectional stigma than non-Latine PWUD (Table 2, Theme 2). CCM staff said the War on Drugs, which disproportionately targeted and penalized people of color with harsher sentences (Alexander, 2012), affected Latine CCM patients as well (e.g., “Latine individuals are oppressed and impacted by mass incarceration and over-policing. We clearly have lots of Latinx patients with SUD with criminal records”). One staff member expressed confusion about the delays in care experienced by Latine adults with criminal justice involvement. The staff member described working with several recently released patients and noted a stark contrast: a non-Latine patient was able to see a provider and receive a prescription for alcohol use disorder immediately after release, while a Latine patient—who was also on dialysis—had to wait over two weeks for a treatment appointment arranged through a federal helpline.

Cultural norms against discussing and disclosing mental health and substance use issues within the Latine community further reinforced negative attitudes against seeking help for SUD. One Latine PWUD refused to seek treatment because he did not want his family to know that he was using substances. Latine females with SUD were subject to traditional gender role expectations that encouraged staying at home, taking a more submissive role, and doing things “a certain way.” The entrenchment of these attitudes was reflected in “this is what you were born to do.” For women, abstinence from substances was an implicit gender-based rule.

### 3.3 Theme 3: structural and interpersonal stigma reinforced each other and shaped individual responses to care

CCM staff accounts described self-stigma, defined as the internalization of negative public attitudes about a stigmatized identity (Link, 1987) (Table 3, Theme 3). Provider mistrust of PWUD who returned to use was internalized by PWUD who said they didn’t “deserve” pain medication because they had returned to using drugs. For example, one CHW noted that despite providers receiving training intended to reduce stigma around substance use, their attitudes remained stigmatizing. In response to perceived mistrust, people who used substances were often not honest with providers.

CCM staff accounts indicated that negative immigrant stereotypes were often internalized by Latine CCM patients. CCM staff described a Latine PWUD who left SUD treatment because he had a court date coming up and anticipated that he would be negatively stereotyped by the judge and sent to jail. Although it was beyond the scope of this study to ask the patient if they internalized this stigma, the fact that they discontinued treatment suggests the power of stereotypes in influencing behavior. In another example, a CCM staff described Latine patients who did not ask for help despite needing food, saying it was “okay” to go without. While people refuse help for many reasons, this individual’s acceptance of personal deprivation suggests an awareness of public stereotypes and perhaps the internalization of immigrant stigma. In other words, because immigrants are negatively stereotyped as burdensome to the system, Latine individuals may not feel worthy of assistance or may feel that others will not want to help them**.** Other Latine PWUD were quoted as saying that they would always remain “drunks”, reflecting a sense of hopelessness in their capacity to change.

CCM staff also noted that some Latine CCM patients would not seek help for mental health or substance use issues because of cultural norms, which were exacerbated by traditional gender roles shaming Latine women for their substance use. According to CCM staff, Latine women with SUD internalized this judgment and refused to seek help: “It takes more to bring Latine women into any kind of help because they have kind of taken in that shame.”

## 4 Discussion

### 4.1 General discussion

The perspectives of CCM staff helped identify stigmatization processes at multiple levels, stigma associated with different aspects of patient identities, and their intersectional nature. CHWs, who help connect hesitant patients to care, can offer unique insights into stigmatization processes as they navigate systems alongside patients. Social structures (societal norms and beliefs), policies, and institutions, produce structural stigma (Link and Phelan, 2001) for PWUD, and Latine PWUD in particular, that was associated with concrete barriers to getting help. Our study adds to the evidence for interpersonal stigma occurring within the context of structural substance use stigma; structural stigma creates an institutional climate in which interpersonal stigma and self-stigma occur. Moreover, as intertwined concepts, interpersonal level stigma processes reinforce structural stigma, further hindering help-seeking. Individual level responses included self-stigma, which could contribute to the under-utilization of substance use treatment services. Finally, individuals with intersectional stigma were described by study participants as experiencing greater barriers to care.

One enactment of structural stigma includes societal norms, policies, and laws that treat members of stigmatized groups differently than the non-stigmatized (Corrigan et al., 2004), thereby communicating that they are less deserving than others (Eskridge and Spedale, 2006). For example, PWUD who returned to substance use often found it difficult to receive medication from suspicious providers, consistent with another study documenting that substance use stigma significantly reduced rates of prescribing pharmacotherapy for people with opioid use disorder (Stone et al., 2021). This difficulty contrasts sharply with how a patient with a chronic medical illness, such as diabetes, is treated if they are not treatment adherent. In another example, the implementation of Section 35 in Massachusetts has been critiqued for not providing sufficient due process to safeguard the same liberties and rights of individuals with SUD that people without SUD take for granted. This contributes to the criminalization of the civil commitment process (Christopher et al., 2020) and of people with SUD (Leis and Rosenbloom, 2009). CCM staff reported witnessing negative interactions between patients and providers around this process. This finding converges with research suggesting that primary care practitioners may have deeper biases against PWUD than psychiatric practitioners (Van Boekel et al., 2013). Our study adds to the growing literature documenting provider and structural stigma as barriers to SUD treatment engagement (Motavalli et al., 2021).

Latine PWUD encountered immigrant stigma, fueled by media depictions of immigrants as illegal trespassers taking advantage of health care and social welfare systems (Brown et al., 2018, Alexander, 2012). Structural stigma can manifest as the lack of inclusion in laws or policies that protect others, which are also used to justify the exclusion of undocumented citizens from basic needs (Link and Phelan, 2001), suggesting that immigrants are “less deserving” and “less equal.” CCM staff also noted a lack of Spanish-translated clinical services and providers, reflecting low prioritization of the needs of this working population (Morey, 2018, Viladrich, 2019). Structural stigma was reinforced by stigmatization processes at the interpersonal level. CCM staff accounts revealed that Latine patients were negatively stereotyped and treated as unwelcome, as communicated in comments like “go back to their country”. This finding is consistent with other studies (Ayón et al., 2012, Fleming et al., 2019, Lopez, 2022, Pinedo et al., 2022).

The stigmas encountered by Latine PWUD were compounded by community norms against disclosing mental health needs, consistent with studies documenting stigmatizing attitudes towards mental illness among Latine adults in North and Central America (Brewer et al., 2024). Further, substance use was more stigmatized than psychosis and depression by Latine individuals at all levels of acculturation (Brewer et al., 2024, Flórez et al., 2015). Staying silent about mental health needs might be rooted in the cultural value of *familismo* which refers to the importance of maintaining harmonious relationships and family unity (Mercado et al., 2023) and is considered “central to the standards of acceptable interpersonal behavior” in Latine communities (Mercado et al., 2023, p. 141; Santiago-Rivera et al., 2002). Latine patients with mental health issues or SUD may feel that disclosing the need for help would disrupt family unity and stability and thus stay quiet to maintain harmony in the family.

Traditional gender role expectations for Latine women include *marianismo*, a cultural script that expects Latine women to prioritize their family’s needs over their own, to not speak up to preserve family harmony, and to avoid using substances (Lee et al., 2019, Mercado et al., 2023, Otiniano Verissimo and Grella, 2017). Latine women who use substances are viewed more negatively than males (Lee et al., 2019, Lee et al., 2006), perhaps due to traditional gender role expectations, leading to shame and the refusal to get help.

Our qualitative study helps to fill a gap in the literature by offering preliminary evidence of how structural stigma processes lead to negative health outcomes, drawing on the unique perspectives of care management staff. Exposure to stigma at multiple levels reinforces negative stereotypes that individuals may begin to endorse about themselves, i.e., “self-stigma” (Link, 1987). Studies have shown Latine patients are reluctant to seek any help for their SUD and that policy exclusions have a “chilling” effect, discouraging efforts to seek assistance (Miller et al., 2022). Stigmatized people may also disengage because they expect rejection (Mendoza-Denton et al., 2002). Internalization of stigma indirectly affects health outcomes by hindering help seeking (Krendl and Perry, 2023) and depleting feelings of self-esteem, worth, and self-efficacy to change (Corrigan et al., 2006, Watson et al., 2007).

### 4.2 Limitations

This study was limited to the views of staff who worked with patients in a region of the U.S., therefore cannot be generalized to Latine adults, who are not a homogeneous identity, or to other parts of the U.S. (Brewer et al., 2024). Future studies should explore the impact of structural stigma from the perspectives of Latine individuals with SUD, accounting for cultural orientation, country of origin, and level of education (Brewer et al., 2024, Flórez et al., 2015). Such investigations can help identify the strengths of stigmatized groups in addressing oppressive systems (Logie et al., 2021). A review of studies done in other countries revealed that CHWs themselves may bear stigmatizing attitudes towards people with mental illness (Carrara et al., 2023). Future studies could focus specifically on their perspectives towards substance use. Additionally, our use of the word "identity" does not account for how Latine patients may self-identify. We aimed to conduct interviews with neutrality but did not explicitly examine how our identities and backgrounds might influence the research process. Future research should place greater emphasis on this aspect to achieve a more balanced interpretation of participant accounts. Finally, although two criteria were met to suggest thematic saturation: exceeding the minimum sample size (> 12 participants) (Bertaux, 1981, Guest et al., 2006, Kuzel, 1992), and the absence of new codes created after incorporating additional participants, the use of purposive sampling would have helped to establish thematic saturation more definitively (Guest et al., 2006). Our study was conducted during the COVID pandemic so although we were able to interview nearly half of those in CCM, participation rates may have been limited.

## 5 Conclusions

Our study extends the structural stigma framework of stigmatized identities and statuses to substance use and ethnicity (Hatzenbuehler, 2016). We found that structural forms of stigma interacted with multiple axes of social stratification (e.g., Latine ethnicity, gender), restricting access to SUD care and perpetuating health disparities (Krendl and Perry, 2023). Messaging at all levels of stigma reinforced the notion that Latine patients did not deserve care. This messaging was reinforced not only within institutions but also across the health care and legal systems. This was exacerbated by bias in the health care system regarding the treatment of substance use and gender bias within the Latine community. As in past studies, the findings indicated that intersectional identities increased barriers to accessing and completing SUD treatment (Benz et al., 2019, Smith et al., 2016).

Our study also suggests links between structural stigma and individual level behaviors in a culturally diverse population (Hatzenbuehler, 2016, Adkins-Jackson et al., 2021). Findings suggest potential pathways through which structural stigma “gets under the skin” to create adverse health outcomes: (Richman and Lattanner, 2014): by creating barriers to seeking and getting help, and by activating self-stigma, which in turn discourages help-seeking behavior (Corrigan et al., 2006, Link and Phelan, 2001). Latina women with SUD, subject to sexism, immigrant and substance use stigma, require tailored solutions to engage them in SUD care.

Understanding beliefs about substance use stigma at multiple levels is important for informing the development of effective interventions. CCM staff, mainly CHWs, suggested interventions at the structural level, such as incentivizing employers to hire individuals with SUD and criminal records, increasing the representation of Spanish-bilingual staff in SUD treatment programs, and offering more training on behavioral health, substance use and cultural competency to maintain positive changes in provider attitudes and behaviors. Some CHWs noted that their role was not to provide specialized care in substance use and that support from other professions was needed, while others felt ill-equipped to discuss SUD with patients. This highlights the need for research on investing in CHWs, a workforce that can have a particularly profound impact on reducing stigma among patients. Our study results point to the need to empirically test how interpersonal level stigma interacts with structural stigma affect health: provider bias, in tandem with policies and laws that restrict access to SUD treatment, may strongly discourage motivation to seek help. This might be particularly applicable in the case of Latine individuals with SUD because of the additional stigma ascribed to immigrant status. These findings underscore the importance of reducing stigma around substance use to encourage help-seeking behaviors. The compounding effects of multiple stigmatized identities can further marginalize at-risk groups, making it crucial to address these challenges through thoughtful policies that ensure access to necessary support.

## CRediT authorship contribution statement

**Mueser Kim T.:** Writing – review & editing. **Rohsenow Damaris J.:** Writing – review & editing, Validation. **Martin Rosemarie:** Writing – review & editing. **Pace Christine A.:** Writing – review & editing, Resources, Formal analysis. **Lopez Victoria:** Writing – review & editing, Project administration, Formal analysis, Data curation. **Colby Suzanne M.:** Writing – review & editing. **Morgan Jake R.:** Writing – review & editing. **Morris Melanie:** Writing – review & editing, Writing – original draft, Formal analysis. **Drainoni Mari-Lynn:** Writing – review & editing, Methodology, Formal analysis. **Kriegsman Ari:** Writing – review & editing. **Cordova-Ramos Erika G.:** Writing – review & editing, Validation, Formal analysis. **Lee Christina S.:** Writing – review & editing, Writing – original draft, Project administration, Investigation, Funding acquisition, Conceptualization.

## Funding

This work was supported by the 10.13039/100000002National Institutes of Health (R01AA028507).

## Declaration of Competing Interest

The authors declare that they have no known competing financial interests or personal relationships that could have appeared to influence the work reported in this paper.
