# Supplementary material for: Care management staff perspectives on stigma and barriers to substance use treatment experienced by latine adults who use substances
Source: Drug Alcohol Depend Rep. 2025 May 10;15:100342. doi: 10.1016/j.dadr.2025.100342 (PMC12148818; doi:10.1016/j.dadr.2025.100342)
Supplement: Supplementary file 1 — Supplementary material [file mmc1.docx]

**Community-Health Worker Version**

- 1. When you have a patient with unhealthy substance use who doesn’t think they have a problem, how do you approach them/how would you raise the issue? How is your approach the same or different for your Latino/a patients?
  2. If the approach is not the same – what are the barriers (cultural, language, stigma)?
     1. What would help you to raise the issue?
  3. What do you think Latinx CCM patients with unhealthy substance use need? What do you think CCM patients with unhealthy substance use need?
     1. What options are available them (Latine, non-Latine) to them as CCM patients? Are these options known to many people and easily accessible?
  4. In working with Latino CCM patients with unhealthy substance use, what are the challenges they (the patients) describe in getting the help they need?
     1. In working with CCM patients in general with unhealthy substance use – what are the challenges they describe
     2. Optional probe: What is your level of awareness of where patients who screen positive for drugs/alcohol go – what is the level of awareness about stigma around getting help for treatment among users themselves
  5. What kind of support or actions might leaders in the CCM take to help Latino CCM patients with unhealthy substance use? CCM patients in general?
  6. How would you describe the CCM organizational climate for patients with unhealthy substance use? Would you say the climate is the same or different for Latino patients with unhealthy SUD? A positive organizational climate is indicated by easily understood policies on how to treat people with SUD.

**Non-Community Health Worker Version**

1. What do you think CCM patients with unhealthy substance use need? How do you think the needs of CCM Latinx patients with unhealthy substance use may be different?
   1. What options are open to them as CCM patients? As Latino CCM patients? Are they widely known and easily accessible for CCM and for Latino CCM patients?
2. In working with Latino CCM patients with unhealthy substance use, what are the challenges they (the patients) describe in getting the help they need?
   1. In working with CCM patients in general with unhealthy substance use – what are the challenges they describe
   2. *Optional probe:* What is your level of awareness of where patients who screen positive for drugs/alcohol go – what is the level of awareness about stigma around getting help for treatment among users themselves
3. What kind of support or actions might leaders in the CCM take to help Latino CCM patients with unhealthy substance use? CCM patients with unhealthy substance use?
   1. Probe – their interest in support/actions
4. How would you describe the CCM organizational climate for patients with unhealthy substance use? Would you say the climate is the same or different for Latino patients with unhealthy SUD? A positive organizational climate is indicated by easily understood policies on how to treat people with SUD.
